# Unlocking Predictive Power: Quantitative Assessment of CAR-T Expansion with Digital Droplet Polymerase Chain Reaction (ddPCR)

**DOI:** 10.3390/ijms25052673

**Published:** 2024-02-26

**Authors:** Eugenio Galli, Marcello Viscovo, Federica Fosso, Ilaria Pansini, Giacomo Di Cesare, Camilla Iacovelli, Elena Maiolo, Federica Sorà, Stefan Hohaus, Simona Sica, Silvia Bellesi, Patrizia Chiusolo

**Affiliations:** 1 Dipartimento di Diagnostica per Immagini, Radioterapia Oncologica ed Ematologia, Fondazione Policlinico Universitario A. Gemelli IRCCS, Largo A. Gemelli 8, 00168 Rome, Italy; 2 Sezione di Ematologia, Dipartimento di Scienze Radiologiche ed Ematologiche, Università Cattolica del Sacro Cuore, 00168 Rome, Italy

**Keywords:** CAR-T cells, digital droplet PCR, B-cell lymphoma

## Abstract

Flow cytometry (FCM) and quantitative PCR (qPCR) are conventional methods for assessing CAR-T expansion, while digital droplet PCR (ddPCR) is emerging as a promising alternative. We monitored CAR-T transcript expansion in 40 B-NHL patients post-infusion of CAR-T products (axi-cel; tisa-cel; and brexu-cel) with both His-Tag FCM and ddPCR techniques. Sensitivity and predictive capacity for efficacy and safety outcomes of ddPCR were analyzed and compared with FCM. A significant correlation between CAR-T counts determined by FCM and CAR transcripts assessed by ddPCR (*p* < 0.001) was observed. FCM revealed median CD3+CAR+ cell counts at 7, 14, and 30 days post-infusion with no significant differences. In contrast, ddPCR-measured median copies of CAR-T transcripts demonstrated significant lower copy numbers in tisa-cel recipients compared to the other products at day 7 and day 14. Patients with a peak of CAR transcripts at day 7 exceeding 5000 copies/microg gDNA, termed “good CAR-T expanders”, were more likely to achieve a favorable response at 3 months (HR 10.79, 95% CI 1.16–100.42, *p* = 0.036). Good CAR-T expanders showed superior progression-free survival at 3, 6, and 12 months compared to poor CAR-T expanders (*p* = 0.088). Those reaching a peak higher than 5000 copies/microg gDNA were more likely to experience severe CRS and ICANS. DdPCR proves to be a practical method for monitoring CAR-T expansion, providing quantitative information that better predicts both treatment outcomes and toxicity.

## 1. Introduction

The advent of CD19-directed chimeric antigen receptor (CAR) T-cell therapy including tisagenlecleucel (tisa-cel), axicabtagene ciloleucel (axi-cel), and brexucabtagene autoleucel (brexu-cel), has marked a significant milestone in the treatment landscape for aggressive B-cell-derived lymphomas. Noteworthy distinctions exist among these therapeutic products encompassing variances in costimulatory domain (CD28 vs. 4-1BB) and indications spanning diffuse large B-cell lymphoma (DLBCL), primary mediastinal B-cell lymphoma (PMBL), mantle cell lymphoma (MCL), and follicular lymphoma (FL).

Compelling data from pivotal trials and real-life experiences indicate a durable complete response (CR) rate from 30% to 40%, with particularly favorable outcomes observed in individuals manifesting an early good response [1]. However, the efficacy of CAR-T therapies is tempered by notable adverse events, primarily cytokine release syndrome (CRS) and immune effector cell-associated neurotoxicity syndrome (ICANS) [2].

CAR-T cells are typically infused intravenously following a short lymphodepleting regimen. The subsequent expansion phase, culminating in peak peripheral blood (PB) concentrations during the second week post-infusion, underscores the dynamic nature of CAR-T cell kinetics [3]. Recent investigations highlight a correlation between CAR-T cell expansion and persistence with a clinical outcome, as well as CAR-T specific toxicities [4,5].

In light of these considerations, establishing a reliable detection strategy for CAR-T cells becomes imperative. Such a strategy holds the potential to predict toxicities or anticipate CAR-T therapy failures, paving the way for tailored subsequent interventions.

Currently, two techniques, flow cytometry (FCM) and polymerase chain reaction (PCR), are employed to assess CAR-T cell expansion in PB. FCM, with its broad applications, facilitates the characterization of CAR-T cell subpopulations, the assessment of effector cell activation and cytotoxic killing, and the quantification of the expression of immunosuppressive molecules [6].

PCR techniques, including both quantitative (qPCR) and digital droplet (ddPCR) assays, offer means to measure the frequency of integrated CAR vectors. qPCR, relying on target specific primers and fluorescent probes, exhibits mild operator dependence and involves continuous enzymatic expansion with specific primers. The transgene copy number per reaction is calculated using a calibration curve and normalized to the copy number of an internal control gene [7]. Digital droplet PCR, known for its sensitivity in detecting low levels of target sequences, has found applications in various contexts, including micro-chimerism analysis, measurable residual disease (MRD), or liquid biopsies [8,9,10].

The year 2020 witnessed the introduction of a novel approach by Mika et al. for the detection and relative quantification of CD19-directed CAR-T in patients treated with axi-cel, as described in more detail later in this text [11]. Moreover, Lou et al. demonstrated the superiority of ddPCR assays compared to qPCR when measuring diluted CAR DNA standards, with a lower detection limit (LoD) of 3.2 transgene copies per milliliter, surpassing the capabilities of qPCR in detecting minute amounts of copies [12]. Specific LoD values for CAR-DNA-directed ddPCR were reported by Badbaran et al., indicating 1–2 and 2–3 copies per 10,000 cells for axi-cel and tisa-cel, respectively [13].

### Aim

The aim of our study was to validate ddPCR as a valuable technique for assessing CAR-T cell expansion in aggressive B-cell lymphomas, comparing it with the current standard of flow cytometric analysis. Additionally, our aim was to explore whether the detection of CAR-T expansion through ddPCR could provide information on CAR-T-specific toxicities and outcomes.

## 2. Results

### 2.1. Patients

During the study period, a total of 57 patients underwent CAR-T therapy. Among those, 17 patients were excluded from the analysis: four had a diagnosis other than lymphoma, and 13 had no evaluable PB samples. This resulted in 40 patients who were included in the study.

The median age of the participants was 59 years, with a range of 28 to 75 years. The majority of patients (73%) presented with diffuse large B-cell lymphoma, had received a median of two previous lines of therapy, and were predominantly treated with axi-cel (50%). Patients did not acknowledge relevant neurologic or cardiac comorbidities, and organ function was adequate in all cases. Overall, all patients previously received treatments with anti CD-20 monoclonal antibodies and anthracyclines-containing regimens, while 17 (42%) patients had received a previous autologous hematopoietic stem cell transplantation. Detailed characteristics of the patients are shown in Table 1.

### 2.2. CAR-T Expansion

**Figure 1:**
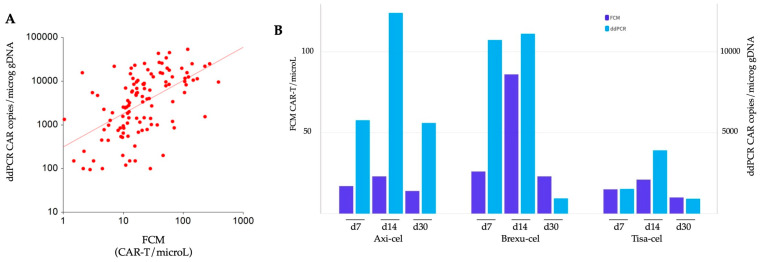
Comparison of CAR-T expansion monitored by flow cytometry (FCM) and digital droplet PCR (ddPCR). (**A**) Spearman correlation analysis: the correlation between the data obtained by FCM and ddPCR is represented using a logarithmic scale on both axes. (**B**) Temporal comparison of expansion: the expansion dynamics of axi-cel, brexu-cel, and tisa-cel at d7, d14, and d30 post CAR-T infusion are illustrated. Dark blue bars depict expansion detected with FCM, while light blue bars represent expansion detected by ddPCR.

We assessed CAR-T expansion at predefined time points at d7, d14, and d30 post-infusion, utilizing two methods, FCM and ddPCR. Data were subsequently compared among different CAR-T products, and the two techniques were also compared. A comprehensive analysis of a total of 120 samples revealed a robust correlation between the two methodologies according to Spearman’s rank test, rho = 0.58, (*p* < 0.001) (Figure 1A).

Both methods revealed a similar kinetic of CAR-T expansion, with peak levels at d14. In FCM, we found a median of 17, 28, and 14 CD3+CAR+ cells/microL measured at d7, d14, and d30, respectively. The peak expansion for all the three products occurred at d14, with 23, 86, and 21 median cells/microL for axi-cel, brexu-cel, and tisa-cel, respectively (Figure 1B). Notably, brexu-cel exhibited a significantly higher CAR-T peak at d14 (*p* = 0.021) compared to the others, while no difference in FCM-detected expansion was found among the three products at d7 and d30 (Supplementary Table S1).

In the analysis of CAR transcripts using ddPCR, median copies of the CAR construct were 3500, 8395, and 1246 copies/microg gDNA at d7, d14, and d30, respectively. The peak expansion was reached at d14 for all products, with 12395, 11109, and 3910 median CAR copies/microg gDNA, respectively (Figure 1B). Tisa-cel resulted in significantly lower expansion than the other products at d7 (*p* = 0.047) and d14 (*p* = 0.031) (Supplementary Table S1).

Notably, no difference was observed among the products at d30 after CAR-T cell infusion.

### 2.3. CAR-T Expansion and Response Outcomes

Efficacy outcomes in terms of response at M3 and PFS were subsequently analyzed, with specific focus on the role of CAR-T expansion as a predictor of response.

The median follow-up for patients was 6.3 months. Of the total cohort, 32 patients could be evaluated for responses at M3 or died of progression before the M3 assessment, while 8 patients were still alive but had not reached the M3 time point at the time of data analysis. At M3, 19 (60%) patients achieved a response (CR or PR), while 13 (40%) patients experienced progression or died of lymphoma.

**Figure 2:**
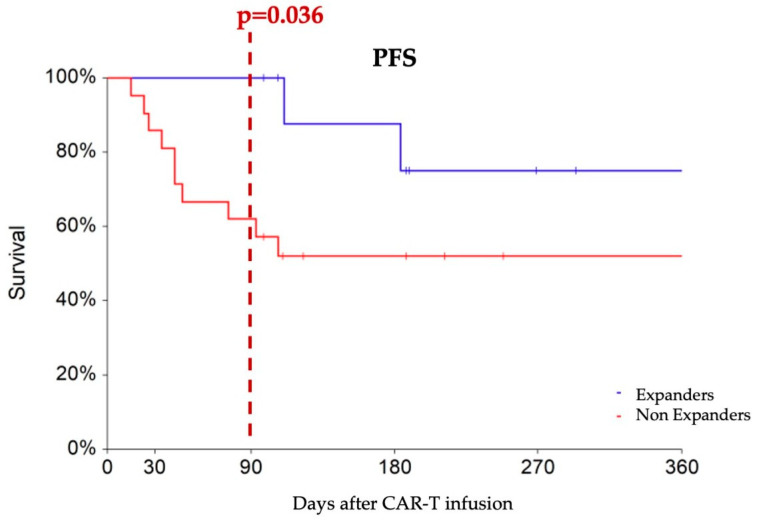
Progression-free survival comparing patients with at least 5000 CAR copies/microg gDNA at day 7 (designated as expanders, blue line) with non-expanders, (red line). At three months after CAR-T infusion, expanders show a higher rate of partial or complete responses compared to non-expanders (100% vs. 62%).

To predict M3 outcomes, we applied ROC analysis to identify a cut-off to the early ddPCR-detected expansion of CAR constructs at d7. Notably, a cutoff value of 5000 copies/microg gDNA emerged as the optimal discriminator between M3 responders and non-responders (AUC 63% *p* = 0.099), demonstrating a sensitivity of 52% and specificity of 92%. Logistic regression analysis revealed that patients with d7 CAR-T transcript levels higher than 5000 copies/microg gDNA—designated as “expanders”—had an increased probability of achieving an M3 response compared to non-expanders (HR 10.79, 95% CI 1.16–100.42, *p* = 0.036). Expanders experienced progression-free survival rates of 100%, 87%, and 75% at 3, 6, and 12 months, respectively, which showed a tendency toward superiority compared to non-expanders (PFS rates of 62% and 52% at the corresponding time points, *p* = 0.088) (Figure 2).

### 2.4. CAR Transcript Expansion and Toxicities

A total of 37 (92.5%) patients experienced CRS of any grade, with a detailed breakdown revealing that 9 (22.5%), 26 (65%), and 2 (5%) patients encountered CRS of grade 1, 2, and 3, respectively. Patients with an overall maximal expansion peak exceeding 5000 copies/microg gDNA were more likely to experience severe CRS (*p* = 0.039, OR 2.05 95% C.I. 10.3–4.08). However, analysis of the CAR expansion at single time points, such as d7 did not exhibit significant differences between patients with maximal CRS graded 0–1 compared to those with CRS graded 2–4 (median 2035, 95% CI 450–22,000, vs. 4177 copies/microg gDNA, 95%CI 1540–11,374, *p* = 0.525) (Supplementary Figure S1). The identified cutoff of 5000 copies/microg gDNA for PFS was not predictive for severe (grade 2 or more) CRS when applied at d7.

Neurotoxicity occurred in 16 (40%) patients, with 3 (7.5%), 5 (12.5%), 6 (15%), and 2 (5%) patients facing ICANS of grade 1, 2, 3, and 4, respectively. Patients with moderate-to-severe (grade 2 or more) ICANS exhibited higher peaks of CAR transcript expansion compared to those with no or mild ICANS (median 6850 copies/microg gDNA, 95% CI 3603–11,650, vs. 15,620 copies/microg gDNA, 95% CI 10,568–25,040, *p* = 0.020). Additionally, patients with an overall maximal CAR transcript expansion higher than 5000 copies/microg gDNA had a higher incidence of grade 2–4 ICANS compared to others (OR 1.46 95% CI 1.05–2.03, *p* = 0.022) (Supplementary Figure S1). This trend was also noticeable when considering the d7 CAR transcript expansion (OR 1.84, 95% CI 0.93–3.66, *p* = 0.079), while we found no correlation with the main markers of inflammation at d7 (see Supplementary Figure S2).

### 2.5. CAR-T FCM-Detected Expansion and Outcomes

In the same cohort, FCM-measured CAR-T peak expansion was tested for prediction of PFS. Neither the expansion at d7 (*p* = 0.729) nor at d14 (*p* = 0.722) predicted PFS in logistic regression analysis. Similarly, we did not demonstrate a strong correlation between FCM-measured CAR-T peak expansion and toxicities. The median CAR-T/microL was 19, 10.8, 11.2, and 66.7/microL when CRS was graded 0, 1, 2, or 3, respectively (*p* = 0.076). When ICANS was graded 0, 1, or 2, the median CAR-T/microL was 17, 11.9, and 14.9/microL respectively (*p* = 0.436).

## 3. Discussion

In this report, we studied the expansion of CAR-T cells post-infusion at predefined time points (d7, d14, and d30) employing and comparing two methods: FCM and ddPCR. Our findings revealed dynamic changes in CAR-T cell levels, with peak expansion consistently occurring at d14 post-infusion. This observation was consistent across the various CAR products and independent from the applied methodology.

Despite the remarkable correlation observed between FCM and ddPCR results in our study, which are in line with previous reports [6,11], it is crucial to consider some technical and conceptual differences between the two methods. FCM aims to enumerate the T cells in PB carrying the CAR transcript on the cell surface. In contrast, DdPCR determines the number of transgene DNA copies of CAR constructs in the total cell population of PB. ddPCR lacks the ability to distinguish expression levels either in the total cell population or on a single-cell basis [6,11].

DdPCR demonstrated a greater sensitivity in detecting low levels of the CAR construct in instances where FCM failed to identify CAR-expressing T cells in PB. However, it is important to note that we cannot entirely rule out the possibility that in samples with positive ddPCR results, the negative FCM results may be attributed to the lack of transcription and surface expression in a significant proportion of cells carrying the transcript. A recent publication reported that when calibrated with house-keeping genes, the estimated proportion between CAR copies and CAR expressing cells may be set around 2–2.5:1 [14].

Similarly, when comparing quantitative PCR (qPCR) and ddPCR, some authors have described differences in the results, even though there is generally good consistency between the two methods. Notably, the mean quantification of CAR obtained with ddPCR may be lower than that obtained with qPCR (approximately 70% if qPCR is set at 100) [12,15].

The three CAR-T products show distinct kinetics in the circulating CAR construct. At d7 and d14, tisa-cel demonstrates lower expansion, with CD28-transduced products showing higher and earlier peaks compared to the 4-1BB-transduced products. In outcome analysis, different products may result in different outcomes, with superior efficacy outcomes possibly observed for CD28-transduced CAR-T, and CAR-T early expansion kinetics may have a role this sense [16]. However, this difference is no longer discernible at d30 following CAR-T infusion. This pattern is in line with previous reports comparing axi-cel and tisa-cel [5,17]. In our experience, the early difference at d7 could be clearly identified with ddPCR, but not with FCM. When plotting data from FCM and ddPCR, we observed that for lower counts in FCM events, the ddPCR seemed to better stratify the expansion with a vast range of CAR copies/microg gDNA values corresponding to relatively limited variations in cells/microL by FCM (Figure 1A). This higher sensitivity may explain why, even in a limited cohort of patients, we were able to discern differences in CAR-T products expansion at d7 with ddPCR, but not with FCM.

We have noted that a higher early expansion measured with ddPCR and defined by at least 5000 CAR copies/microg gDNA at d7 could predict a favorable clinical outcome. Individuals meeting the criteria for early expansion were more likely to achieve a response at M3. This observation aligns with other studies, where patients exhibiting robust early expansion detected by FCM at day 10 achieved better outcomes compared to non-expanders [5,18].

Interestingly, a higher peak of CAR transcript predicts prognosis when determined at d7, but not at d14. In the context of aggressive lymphomas, CAR-T cells may exert their therapeutic effect during the very early phase, rather than in a later period. This observation is in line also with findings described by Frank and colleagues, who determined circulating tumor DNA (ctDNA) pre-lymphodepletion and at various time points after infusion. In their study, patients with durable responses exhibited a very early clearance of ctDNA, with undetectable levels from d7 after CAR-T infusion in 70% of cases [19]. This emphasizes the importance of high sensitivity in early response prognostication techniques.

With regards to CAR-T toxicities, a higher level of CAR transcript was associated with more severe toxicities, in particular in patients surpassing the cut-off value of 5000 CAR copies/microg gDNA who demonstrated a higher risk of grade ≥ 2 CRS or ICANS. Consistent with previous studies, a correlation between high levels of CAR T-cell expansion, high tumor burden, and the occurrence of high-grade CRS and incidence of ICANS has been established.

The advantages of ddPCR assay consist of a rapid (about 2 h), precise, and reliable quantification of the relative abundance of specific transgene sequences. Moreover, the test results are less operator-dependent than qPCR, the analysis costs are lower, and samples can be safely stored by freezing. As obtained from peripheral whole blood, DNA used for ddPCR comes in part from intracellular DNA and in part from cell-free floating DNA as a result of cell death; therefore, one of the main limitations of ddPCR is measuring the average number of vector copies integrated into the entire sample tested, but not providing information on the actual number of circulating CAR-T cells or on their features.

The integration of data from FCM and ddPCR may represent a useful tool in discriminating clinical scenarios [20].

Limitations of this study are the heterogeneity of our patient cohort, which included different histological subtypes (DLBCL, tFL and MCL), and the monocentric study design. Additionally, longer-term follow-up is required to provide further insights into CAR T-cell kinetics and the impact on treatment outcomes.

## 4. Methods and Methods

All patients treated in our center with anti-CD19 CAR-T between September 2019 and September 2023 were retrospectively screened for inclusion in the study. Eligibility criteria included the receipt of CAR-T therapy for an aggressive B-cell lymphoma, an age exceeding 18 years, and the provision of informed consent. Exclusion criteria involved the unavailability of evaluable biological samples for technical reasons or withdrawal of informed consent.

Demographic variables and disease-related characteristics were collected for data analysis. PB samples were obtained from patients at predefined time points, specifically at 7 (d7), 14 (d14), and 30 (d30) days post CAR-T infusion.

CAR-T cell-related toxicities, namely CRS and ICANS, were graded on a scale from 0 to 5 according to the recommendations of the American Society for Transplantation and Cellular Therapy (ASTCT) [2].

Disease outcomes were assessed through 18-FDG PET-CT scans conducted at 1-, 3-, 6-, and 12-months post CAR-T infusion. Responders were identified as patients achieving complete response (CR) or partial response (PR), while non-responders included those with stable disease (SD), progression (PD), or relapse. Progression-free survival (PFS) and overall survival (OS) were calculated from the time of CAR-T infusion.

Peripheral blood samples were established at 7, 14, and 30 days after CAR-T infusion. At each timepoint, 4 milliliters of EDTA-anticoagulated whole blood for FCM and 9 milliliters of EDTA-anticoagulated whole blood for ddPCR were collected from patients.

### 4.1. Digital Droplet PCR (ddPCR)

The ddPCR essays were conducted following the methodology described by Mika and colleagues [11].

For each timepoint, 9 mL of peripheral blood were collected in EDTA. Samples were centrifuged for 5 min at 3500 rpm, and the buffy coat was removed; DNA was subsequently extracted with the QIAmp Blood Mini kit (250) (Qiagen, Hilden, Germany) for the various samples.

The CD19 CAR-T 20X assay is designed to detect CD19 CAR-T DNA from both axi-cel and tisa-cel constructs. This essay must be combined with an HEX copy number assay RPP30. The two assays and the ddPCR Supermix for Probes (No dUTP) mix were brought to room temperature, mixed thoroughly by vortexing, centrifuged briefly to collect the contents at the bottom of each tube, and stored away from the light. Before preparing the reaction mixture, we prepared the samples one by one at a concentration of 50 ng/microL, according to the guidelines. As a negative control, we used a well containing only the mix and H_2_O instead of the sample. For a total volume of 22 microL of MasterMix, we mixed 11 microL of ddPCR Supermix for Probes (No dUTP), 1.1 microL of probe CD-19 CAR-T 20× target (FAM) and RPP30 (HEX), 1 microL H_2_O, and 7.8 microL DNA sample.

A standard volume of 20 mL of reaction mix was composed of 10 mL of 2 ddPCR Supermix (without dUTPs; Bio-Rad, Hercules, CA, USA), 4 mL of fluorescently labeled primers and probes, and 2 mL of DNA template. Digital PCR was performed using the Quantalife QX200 Droplet Digital PCR system (Bio-Rad). The QX200 droplet generator (Bio-Rad) generated droplets in eight-well cartridges. The water-in-oil emulsions were pipette-transferred to a 96-well polypropylene plate (Bio-Rad), which was sealed with foil and placed in an ABI thermal cycler. The mixes were amplified until the endpoint was reached under the following conditions: 95 °C for 5 min, 95 °C for 30 s, 60 °C for 1 min (40 cycles, 2.5 °C/s ramp rate) with a 10 min hold at 98 °C, and a final hold at 4 °C. After PCR, the plate was processed with the QX200 droplet reader (Bio-Rad). According to the manufacturer’s recommendations, the ddPCR results were analyzed using QuantaSoft software version 1.7.4 (Bio-Rad). Each reaction was individually analyzed, and the thresholds were manually adjusted when necessary and adjusted separately for the FAM and VIC channels. The droplet reader software results were represented as copies/mL for CAR, and the CAR copy number/DNA concentration ratio represents the number of CAR-T cells per microgram in total peripheral blood.

For the assay’s specificity, sensitivity, and reproducibility, we referred to Badbran et al. [13]. The authors demonstrated that this assay exhibits excellent specificity (no positive signal observed in multiple samples from non-treated individuals) along with the highest possible sensitivity (detection of single copies). In fact, it ensures the detection of CAR-T cells at concentrations as low as 1–2 axi-cel/brexu-cel or 2–3 tisa-cel per 10,000 blood cells in routine use (i.e., with approximately 100 ng gDNA, corresponding to 15,000 diploid cells).

For the detection range, the upper limit of detection for ddPCR is constrained by the saturation of positive droplets; however, this limitation can be mitigated through sample dilution. CAR copies were quantified and expressed as copy/microg gDNA and copy/microL blood. The copy/microg gDNA values were derived by calculating the ratio of CAR-T to reference gene copy numbers, normalized by the number of DNA per genome copy number.

### 4.2. Flow Cytometry (FCM)

The flow cytometry analysis for CAR-T cell detection was performed in accordance with established protocols outlined in previous publications [21,22]. The total leukocyte count and the absolute count of CD3+, CD4+, and CD8+ lymphocytes were obtained using a single-platform method with a standard antibody cocktail TETRA-1 by AQUIOS cytometer (Beckman Coulter, Brea, CA, USA). The staining volume for the CAR-T detection tube was determined based on the total leukocyte count, with a median value of the staining volume of 200–400 peripheral blood volume microliters. The identification of CAR-T cells was performed through an indirect method with a human recombinant CD19 histidine Tag protein (Acro biosystems cat CD9-H52H2, Newark, DE, USA) in conjunction with an anti-His APC-labelled secondary antibody (eBiolegend cat 362605, San Diego, CA, USA). We performed two surface stainings with the following monoclonal antibodies: Tube1-negative control with CD45 (V-500 clone 2D1); CD3 (FITC clone HIT3a); CD8 (PC7 clone SFC21Thy2D3); CD4 (APC-H7 clone SK3); and Tube2 CAR-T detection tube with CD45, CD3, CD8, CAR-T, and CD4. CD45, CD3, and CD4 monoclonal antibodies were from Becton Dickinson (Franklin Lakes, NJ, USA), while CD8 was from Beckman Coulter.

For flow cytometry CAR detection, peripheral blood was incubated with the CD19-His recombinant protein for 20 min at room temperature. Following incubation, the cells were washed with the buffer containing phosphate-buffered saline (PBS) + 1% HSA and then incubated with the secondary anti-tag APC-conjugated anti-His antibody. After 15 min, cells were washed again and incubated with all the other surface markers based on the panel, as already reported. Cells were incubated for 10 min with ammonium chloride lysing solution (BD Biosciences, Berkshire, UK), centrifuged, and resuspended in PBS for acquisition. A minimum of approximately 200,000 CD45+ events per tube were recorded. Data were acquired with BD FACSCanto II cytometer and analyzed with FACSDiva Software (Becton Dickinson, BD FACSDiva v9.2). Tube 1 was used for setting the positivity of CAR-T cells with a fluorescence minus one (FMO)-negative control. The second tube was performed to define the percentage of CD3+ CAR-T on the total CD3+ lymphocyte population and the percentage of CAR-T CD4+ and CAR-T CD8+. The absolute count of the CAR-T population was defined with a double-platform method.

### 4.3. Statistical Analysis

All variables underwent normality testing, and non-normally distributed variables were assessed using rank-sum tests. Spearman’s test was employed to evaluate correlations between two parametric variables such as ddPCR and FCM. To establish a cut-off value as a risk factor for a dichotomous event, Receiver Operating Characteristic (ROC) analysis was applied, and the Area Under the Curve (AUC) was determined. Comparisons between parametric and categorical variables were analyzed with Student’s t, Kruskal–Wallis, or ANOVA tests. Correlations between categorical variables and binary outcomes (e.g., response at 3 months) were studied using logistic regression analysis, while PFS curves were generated through Kaplan–Meier analysis.

All statistical analyses were performed using the NCSS 2020 Statistical Software by NCSS, LLC, Kaysville, UT, USA.

## 5. Conclusions

Digital droplet PCR is highly applicable in the context of CAR-T. The information obtained agrees with that in FCM and is possibly more sensitive. In the ddPCR method, an early peak expansion is predictive of efficacy and toxicity. DdPCR may represent a valid tool for determination of CAR-T expansion, with some bench advantages in terms of sensitivity and reproducibility.

## Figures and Tables

**Table 1:** Characteristics of patients.

Age	Median	59 (Range 28–75)
Gender	Female	19 (47%)
	Male	21 (53%)
Diagnosis	Diffuse large B-cell lymphoma	29 (73%)
	Primary mediastinal B-cell lymphoma	3 (7%)
	Mantle cell lymphoma	8 (20%)
Previous lines	Median	2 (range 2–6)
IPI score	Median	3 (range 0–5)
LDH *	Normal	20 (50%)
	Elevated	20 (50%)
CAR-T product	Axi-cel	20 (50%)
	Brexu-cel	8 (20%)
	Tisa-cel	12 (30%)
Status at CAR-T infusion **	PD	16 (40%)
	SD	10 (25%)
	PR/CR	14 (35%)

* LDH normal levels were set at 250 UI/L as per upper normality cut-off set at our laboratory. ** The status at CAR-T infusion was determined with PET-CT or total body CT. The definition of progressive disease (PD), stable disease (SD), partial or complete response (PR, CR) is referred to the global clinical evaluation after the bridging therapy.

## Data Availability

Data is contained within the article and Supplementary Materials.

## Supplementary Materials

The supporting information can be downloaded at: https://www.mdpi.com/article/10.3390/ijms25052673/s1.

## References

[1] Vercellino L., Di Blasi R., Kanoun S., Tessoulin B., Rossi C., D’Aveni-Piney M., Obéric L., Bodet-Milin C., Bories P., Olivier P. (2020). Predictive factors of early progression after CAR T-cell therapy in relapsed/refractory diffuse large B-cell lymphoma. Blood Adv..

[2] Lee D.W., Santomasso B.D., Locke F.L., Ghobadi A., Turtle C.J., Brudno J.N., Maus M.V., Park J.H., Mead E., Pavletic S. (2019). ASTCT Consensus Grading for Cytokine Release Syndrome and Neurologic Toxicity Associated with Immune Effector Cells. Biol. Blood Marrow Transplant..

[3] Hirayama A.V., Gauthier J., Hay K.A., Voutsinas J.M., Wu Q., Gooley T., Li D., Cherian S., Chen X., Pender B.S. (2019). The response to lymphodepletion impacts PFS in patients with aggressive non-Hodgkin lymphoma treated with CD19 CAR T cells. Blood.

[4] Wittibschlager V., Bacher U., Seipel K., Porret N., Wiedemann G., Haslebacher C., Hoffmann M., Daskalakis M., Akhoundova D., Pabst T. (2023). CAR T-Cell Persistence Correlates with Improved Outcome in Patients with B-Cell Lymphoma. Int. J. Mol. Sci..

[5] Monfrini C., Stella F., Aragona V., Magni M., Ljevar S., Vella C., Fardella E., Chiappella A., Nanetti F., Pennisi M. (2022). Phenotypic Composition of Commercial Anti-CD19 CAR T Cells Affects In Vivo Expansion and Disease Response in Patients with Large B-cell Lymphoma. Clin. Cancer Res..

[6] Cheng J., Mao X., Chen C., Long X., Chen L., Zhou J., Zhu L. (2023). Monitoring anti-CD19 chimeric antigen receptor T cell population by flow cytometry and its consistency with digital droplet polymerase chain reaction. Cytometry A.

[7] Hu Y., Huang J. (2020). The Chimeric Antigen Receptor Detection Toolkit. Front. Immunol..

[8] Link-Lenczowska D., Pallisgaard N., Cordua S., Zawada M., Czekalska S., Krochmalczyk D., Kanduła Z., Sacha T. (2018). A comparison of qPCR and ddPCR used for quantification of the JAK2 V617F allele burden in Ph negative MPNs. Ann. Hematol..

[9] Franke G.-N., Maier J., Wildenberger K., Cross M., Giles F.J., Müller M.C., Hochhaus A., Niederwieser D., Lange T. (2020). Comparison of Real-Time Quantitative PCR and Digital Droplet PCR for BCR-ABL1 Monitoring in Patients with Chronic Myeloid Leukemia. J. Mol. Diagn..

[10] Mika T., Baraniskin A., Ladigan S., Wulf G., Dierks S., Haase D., Schork K., Turewicz M., Eisenacher M., Schmiegel W. (2019). Digital droplet PCR-based chimerism analysis for monitoring of hematopoietic engraftment after allogeneic stem cell transplantation. Int. J. Lab. Hematol..

[11] Mika T., Maghnouj A., Klein-Scory S., Ladigan-Badura S., Baraniskin A., Thomson J., Hasenkamp J., Hahn S.A., Wulf G., Schroers R. (2020). Digital-Droplet PCR for Quantification of CD19-Directed CAR T-Cells. Front. Mol. Biosci..

[12] Lou Y., Chen C., Long X., Gu J., Xiao M., Wang D., Zhou X., Li T., Hong Z., Li C. (2020). Detection and Quantification of Chimeric Antigen Receptor Transgene Copy Number by Droplet Digital PCR versus Real-Time PCR. J. Mol. Diagn..

[13] Badbaran A., Berger C., Riecken K., Kruchen A., Geffken M., Müller I., Kröger N., Ayuk F.A., Fehse B. (2020). Accurate In-Vivo Quantification of CD19 CAR-T Cells after Treatment with Axicabtagene Ciloleucel (Axi-Cel) and Tisagenlecleucel (Tisa-Cel) Using Digital PCR. Cancers.

[14] de la Iglesia-San Sebastián I., Carbonell D., Bastos-Oreiro M., Pérez-Corral A., Bailén R., Chicano M., Muñiz P., Monsalvo S., Escudero-Fernández A., Oarbeascoa G. (2024). Digital PCR Improves Sensitivity and Quantification in Monitoring CAR-T Cells in B Cell Lymphoma Patients. Transplant. Cell Ther. Epub.

[15] Schubert M.-L., Berger C., Kunz A., Schmitt A., Badbaran A., Neuber B., Zeschke S., Wang L., Riecken K., Hückelhoven-Krauss A. (2022). Comparison of single copy gene-based duplex quantitative PCR and digital droplet PCR for monitoring of expansion of CD19-directed CAR T cells in treated patients. Int. J. Oncol..

[16] Gauthier J., Gazeau N., Hirayama A.V., Hill J.A., Wu V., Cearley A., Perkins P., Kirk A., Shadman M., Chow V.A. (2022). Impact of CD19 CAR T-cell product type on outcomes in relapsed or refractory aggressive B-NHL. Blood.

[17] Fürst D., Neuchel C., Neagoie A., Amann E., Rode I., Krauss A., Schrezenmeier H., Wais V., Döhner H., Viardot A. (2022). Monitoring the In-Vivo Expansion and Persistence of CAR-T Cells As a Tool to Help Decision Making in Patients with Aggressive B-Cell Lymphoma. Blood.

[18] Ayuk F.A., Berger C., Badbaran A., Zabelina T., Sonntag T., Riecken K., Geffken M., Wichmann D., Frenzel C., Thayssen G. (2021). Axicabtagene ciloleucel in vivo expansion and treatment outcome in aggressive B-cell lymphoma in a real-world setting. Blood Adv..

[19] Frank M.J., Hossain N.M., Bukhari A., Dean E., Spiegel J.Y., Claire G.K., Kirsch I., Jacob A.P., Mullins C.D., Lee L.W. (2021). Monitoring of Circulating Tumor DNA Improves Early Relapse Detection After Axicabtagene Ciloleucel Infusion in Large B-Cell Lymphoma: Results of a Prospective Multi-Institutional Trial. J. Clin. Oncol..

[20] Haderbache R., Warda W., Hervouet E., da Rocha M.N., Trad R., Allain V., Nicod C., Thieblemeont C., Boissel N., Varlet P. (2021). Droplet digital PCR allows vector copy number assessment and monitoring of experimental CAR T cells in murine xenograft models or approved CD19 CAR T cell-treated patients. J. Transl. Med..

[21] Zaninelli S., Meli C., Borleri G., Quaroni M., Pavoni C., Gaipa G., Biondi A., Introna M., Golay J., Rambaldi A. (2023). Optimization and validation of in vivo flow cytometry chimeric antigen receptor T cell detection method using CD19his indirect staining. Cytometry A.

[22] Galli E., Bellesi S., Pansini I., Di Cesare G., Iacovelli C., Malafronte R., Maiolo E., Chiusolo P., Sica S., Sorà F. (2023). The CD4/CD8 ratio of infused CD19-CAR-T is a prognostic factor for efficacy and toxicity. Br. J. Haematol..

