# Supplementary material for: Unlocking Predictive Power: Quantitative Assessment of CAR-T Expansion with Digital Droplet Polymerase Chain Reaction (ddPCR)
Source: Int J Mol Sci. 2024 Feb 26;25(5):2673. doi: 10.3390/ijms25052673 (PMC10932155; doi:10.3390/ijms25052673)

SUPPLEMENTARY

SUPPLEMENTARY TABLE S1

Supplementary Table S1 : CAR-T expansion in peripheral blood according to type of CAR-T product , utilizing FCM and ddPCR at predetermined time points.

	Day 7		Day 14		Day 30	
	FCM	ddPCR	FCM	ddPCR	FCM	ddPCR
	CAR-T/microL	copies/microg	CAR-T/microL	copies/microg	CAR-T/microL	copies/microg
Axi-cel	17	5765	23	12395	14	5596
Brexu-cel	26	10722	86	11109	23	942
Tisa-cel	15	1523	21	3910	10	920
p value	0.903	0.047	0.021	0.031	0.146	0.254

Supplementary Figure S1

In supplementary Figure S1, CAR copies detected ad D7 are represented in association with the maximal CRS (any grade-(**A**)- and grouped 0-1 vs 2-4-(**B**)), showing a trend for increased CRS with growing expansion, despite this is not statistically relevant. This may be due to the fact that the median onset of CRS is normally earlier, mostly between day 2 and 4. In (**C**,**D**) the CAR-T expansion at D7 is compared to maximal ICANS grade (any grade and grouped 0-1 vs 2-4): in this case, greater expansion correlates with worse ICANS.

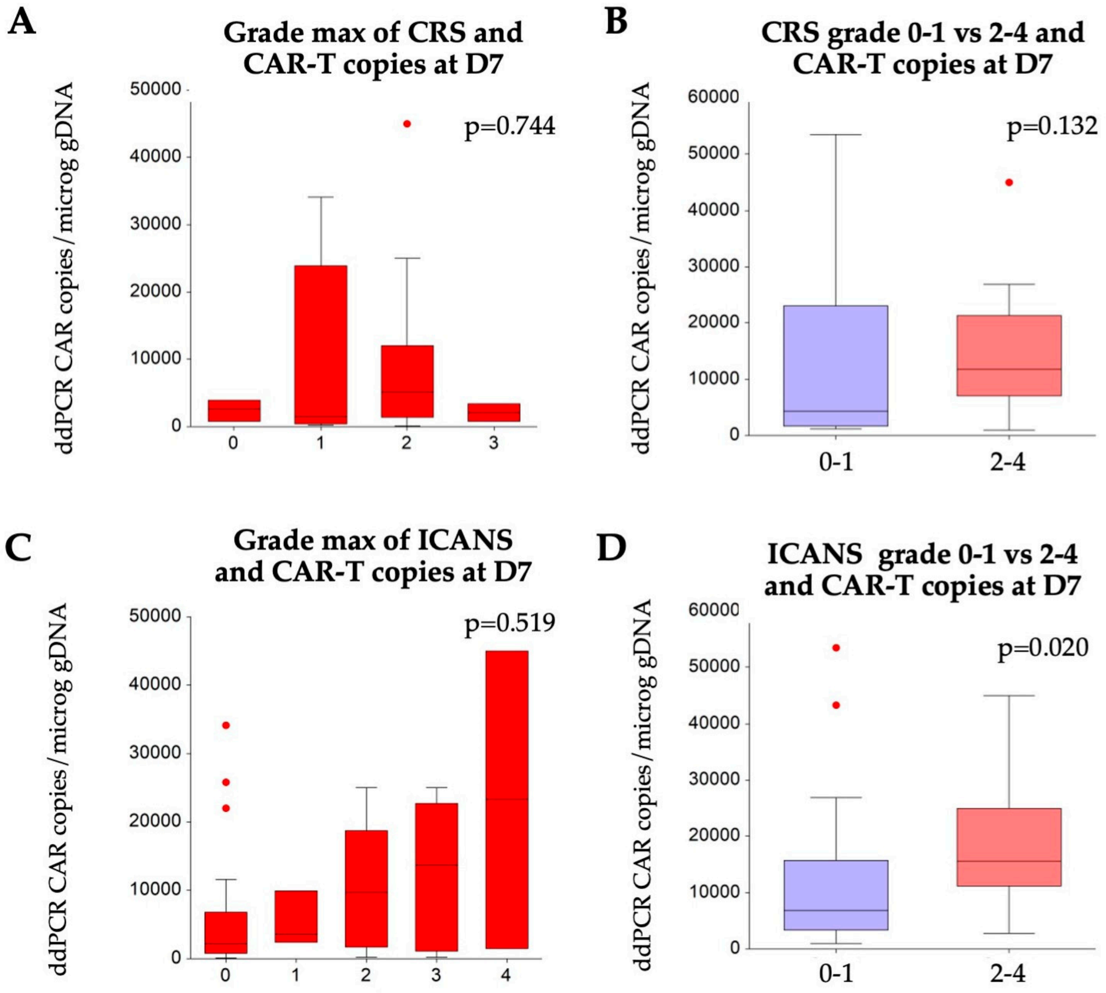

Supplementary Figure S2

In supplementary Figure S1, CAR copies detected ad D7 are represented in association with the main inflammatory markers at day 7, namely C-reactive protein, interleukin 6, and receptor of interleukin 2. Measured at day7, we found no strong association between CAR transcript and inflammation markers. This may be due to earlier onset of CRS compared to scheduled day7 sampling.

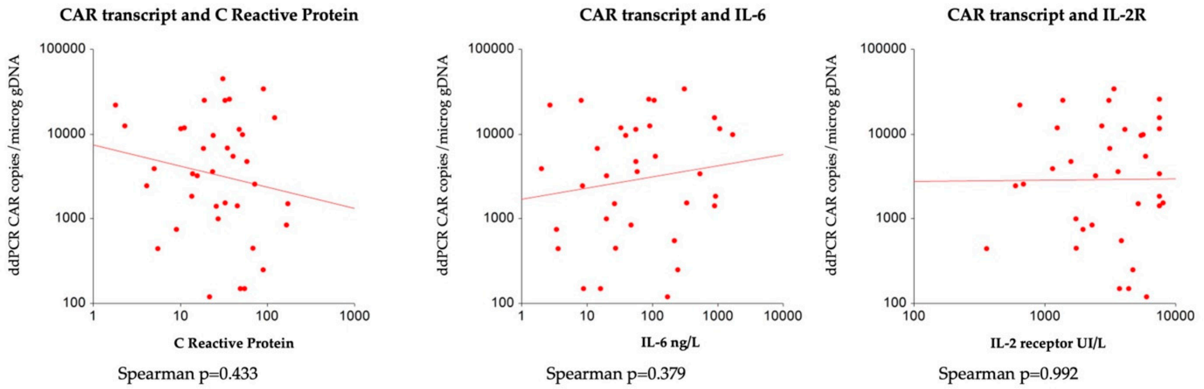

Supplement: Supplementary file 1 [file ijms-25-02673-s001.zip › ijms-2849666-supplementary.pdf]
